# River barriers and cryptic biodiversity in an evolutionary museum

**DOI:** 10.1002/ece3.482

**Published:** 2013-01-25

**Authors:** G Voelker, B D Marks, C Kahindo, U A'genonga, F Bapeamoni, L E Duffie, J W Huntley, E Mulotwa, S A Rosenbaum, J E Light

**Affiliations:** 1 Department of Wildlife and Fisheries Sciences, Texas A&M UniversityCollege Station, 77843, USA; 2 Université Officielle de Bukavu, Faculté des Sciences, Département de BiologieBukavu, B.P. 570, Democratic Republic of the Congo; 3 Laboratoire d'Ecologie et de Gention des Ressources Animales, Faculté des Sciences de l'Univerité de KisanganiKisangani, B.P. 2012, Democratic Republic of the Congo

**Keywords:** Afrotropics, birds, Congo River, cryptic diversity, lice, river barriers

## Abstract

The Riverine Barriers Hypothesis (RBH) posits that tropical rivers can be effective barriers to gene flow, based on observations that range boundaries often coincide with river barriers. Over the last 160 years, the RBH has received attention from various perspectives, with a particular focus on vertebrates in the Amazon Basin. To our knowledge, no molecular assessment of the RBH has been conducted on birds in the Afrotropics, despite its rich avifauna and many Afrotropical bird species being widely distributed across numerous watersheds and basins. Here, we provide the first genetic evidence that an Afrotropical river has served as a barrier for birds and for their lice, based on four understory bird species collected from sites north and south of the Congo River. Our results indicate near-contemporaneous, Pleistocene lineage diversification across the Congo River in these species. Our results further indicate differing levels of genetic variation in bird lice; the extent of this variation appears linked to the life-history of both the host and the louse. Extensive cryptic diversity likely is being harbored in Afrotropical forests, in both understory birds and their lice. Therefore, these forests may not be “museums” of old lineages. Rather, substantial evolutionary diversification may have occurred in Afrotropical forests throughout the Pleistocene, supporting the Pleistocene Forest Refuge Hypothesis. Strong genetic variation in birds and their lice within a small part of the Congo Basin forest indicates that we may have grossly underestimated diversity in the Afrotropics, making these forests home of substantial biodiversity in need of conservation.

## Introduction

Alfred Russel Wallace was the first to hypothesize that tropical rivers are effective barriers to animal distributions (Wallace 1852). This hypothesis stemmed from his observations that range boundaries often abut at rivers, suggesting that rivers are barriers to gene flow. If true, genetic divergence (population structure) should be evident across river barriers, which could ultimately result in the formation of new species where rivers serve as boundaries between sister taxa. Over the last 160 years, the Riverine Barriers Hypothesis (RBH) has received attention from various perspectives, particularly in the Amazon Basin where Wallace made his initial observations on primates (Capparella 1988; Ayres and Clutton-Brock 1992; Colwell 2000). In the Amazon Basin, support for the RBH has come from intraspecific molecular systematic studies of birds (Capparella 1988; Bates et al. 2004; Cheviron et al. 2005; Aleixo 2006; Ribas et al. 2012) and other vertebrate taxa (Lampert et al. 2003; Pellegrino et al. 2005; Vallinoto et al. 2006). However, it is important to note that vicariant forces other than rivers (e.g., tectonic events) are often implicated as factors promoting lineage diversification and distributions that seemingly support the RBH (Patton et al. 1994; Haffer 1997; Lougheed et al. 1999; Gascon et al. 2000).

In contrast to the study of Amazonian taxa, molecular systematic assessments of the RBH for vertebrate taxa in the extensive tropical forests of west and central Africa has been largely limited to studies of primates and rodents distributed across Guineo-Congolean (Afrotropical) watersheds and river basins (Quérouil et al. 2003; Telfer et al. 2003; Anthony et al. 2007; Kennis et al. 2011; Nicolas et al. 2011). The limited support for the RBH in these taxa has been largely confined geographically to divergence across the Sanaga and Ogooue Rivers (Quérouil et al. 2003; Telfer et al. 2003; Anthony et al. 2007; Nicolas et al. 2011). These rivers are relatively short (890 and 1200 km, respectively), and generally restricted to Cameroon (Sanaga) and Gabon (Ogooue). To our knowledge, no molecular assessment of the RBH has been conducted on birds in the Afrotropics, despite its rich avifauna and with many Afrotropical bird species being, like rodents and primates, widely distributed across numerous watersheds and basins (Sinclair and Ryan 2010).

The paucity of assessments of the RBH in Afrotropical birds is thus surprising, particularly considering the size of major rivers. This might reflect the general absence of intraspecific plumage variation in birds distributed in Afrotropical forests, and the idea that this reflects an absence of genetic variation as well. Indeed, this lack of plumage variation led Mayr and O'Hara (Mayr and O'Hara 1986) to discount as uninformative fully half of all lowland forest birds in their assessment of the Pleistocene Forest Refuge Hypothesis. This was taken a step further by Fjeldså and Lovett (Fjeldså and Lovett 1997), who labeled Afrotropical lowland forests as being “museums” of old lineages, implying that evolutionary processes had, in essence, ground to a halt there. An additional confounding issue is that most avian collections from the Congo Basin are relatively old (1970′s and earlier), and thus there is a general lack of well-preserved DNA for genetic analysis.

**Figure 1:**
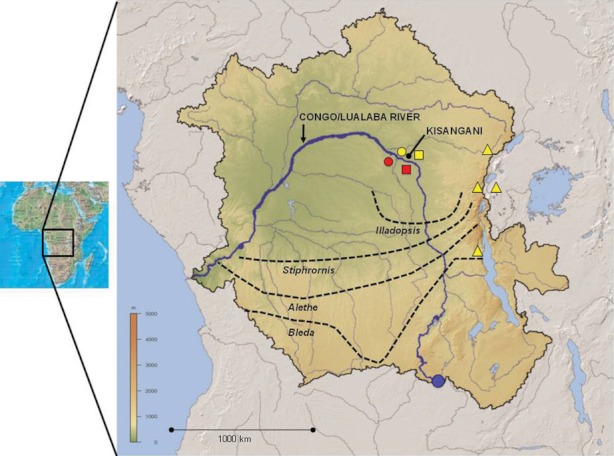
The Congo River basin, with major tributaries. The blue circle indicates the location of the Congo River headwaters. Dashed lines denote southernmost extent of species' distributions. Our sampling sites near Kisangani are denoted by red (south of the Congo River) and yellow (north) symbols, with different shapes to reflect sampling sites on opposite sides of Congo tributary rivers. Yellow triangles indicate localities for additional samples included in analyses.

To our knowledge, there have been no intraspecific studies of genetic variation assessing the RBH in vertebrate taxa across the Congo-Lualaba River (Congo River hereafter). The Congo River flows 4372 km, 80% of which is between 567 and 293 m in elevation, and ranges up to 15 km in width (Runge 2007). It is thus the longest and widest Afrotropical river and for much of its course, the river severs the extensive western and central Afrotropical forests from a comparatively small area of that forest in the southern Democratic Republic of the Congo. Importantly, the Congo headwaters are found in savannahs, over 1000 km south of tropical forest habitat (Runge 2007). Further, the Congo Basin was effectively a large lake until the latest Pliocene-early Pleistocene when it was drained to the Atlantic via river capture (Beadle 1981; Stankiewicz and de Wit 2006). The river system (Congo and tributaries; Fig. 1) has been in continuous existence since that time, and has experienced little change related to tectonic or climate factors (Beadle 1981). These combined features of the Congo River suggest, (1) that the river is a potentially formidable, stable barrier to gene flow in tropical forest lineages; (2) the lack of habitat connectivity around river headwaters will likely decrease genetic connectivity and is thus unlikely to explain divergences on either side of the river in those lineages; and (3) that divergences across the river should have occurred relatively recently in evolutionary time.

Here, we report results from a taxonomically diverse suite of bird species, six of which are forest understory specialists: *Alethe castanea* (Fire-crested Alethe), *Bleda syndactylus* (Red-tailed Bristle-bill), *Illadopsis rufipennis* (Pale-breasted Illadopsis), *Stiphrornis xanthogaster* (Eastern Forest Robin), *Neocossyphus poensis* (White-tailed Rufous Thrush) and *Bleda ugandae* (Yellow-eyed Bristle-bill)*,* and four that are forest habitat generalists: *Terpsiphone rufiventer* (Red-bellied Paradise Flycatcher), *Criniger calurus* (Red-tailed Greenbul), *Myioceyx leconteii* (African Dwarf-Kingfisher) and *Smithornis rufolateralis* (Rufous-sided Broadbill). Our results provide the first genetic evidence that an Afrotropical river has served as a barrier to gene flow for four understory specialist bird species and their lice based on samples collected from sites north and south of the Congo River.

## Materials and Methods

Our field sampling was conducted in 2010 and 2011. In 2010, we sampled both north and south of the Congo River, near Kisangani (Fig. 1). In 2011, we again sampled north and south of the Congo River, but samples also were collected on opposite sides of Congo River tributaries to increase potential across-river comparisons. On the north bank, we collected west of the Lindi River, whereas on the south bank, we collected west of the Lomami River. Birds were collected via mist nets and euthanized following approved protocols (Texas A&M University Animal Use Protocol No. 2009-028). Collection sites and bird species included in this study are presented in Table 1; the variation in numbers across species is indicative of their population densities. For both *Bleda syndactyla* and *Stiphrornis xanthogaster*, we also included preserved tissues samples collected by the Field Museum of Natural History (FMNH) from areas north and well to the east of the Congo River (triangles in Fig. 1).

**Table 1:** General sampling localities and sample sizes for species analyzed

	South Bank of Congo River		North Bank of Congo River	
		
	West of Lomami River	East of Lomami River (Kisangani)	West of Lindi River	East of Lindi River (Kisangani)
Understory species				
*Alethe castanea*	2	7	5	3
*Bleda syndactyla*	3	8	3	10
*Illadopsis rufipennis*	–	7	6	3
*Stiphrornis xanthogaster*	–	8	3	10
*Neocossyphus poensis*	–	4	–	1
*Bleda ugandae*	–	6	–	6
Habitat generalists				
*Criniger calurus*	–	5	–	3
*Myioceyx leconteii*	–	3	–	2
*Terpsiphone rufiventer*	–	5	–	2
*Smithornis rufolateralis*	–	1	–	1

Birds were prepared in the field as standard museum skins, with associated tissue and blood samples. Prior to preparation, birds were exposed to ethyl acetate and brushed for louse ectoparasites. Birds were identified to species and lice to genus using morphology and both have been catalogued into the Texas Cooperative Wildlife Collection at Texas A&M University. Of the specimens brushed for ectoparasites, there were only three instances where lice were collected from individuals of the same bird species on opposite sides of the Congo River: *Myrsidea* sp. (Phthiraptera: Amblycera) parasitizing *Alethe castanea*, *Myrsidea* sp. parasitizing *Terpisphone rufiventer* and *Sturnidoecus* sp. (Phthiraptera: Ischnocera) parasitizing *Terpsiphone rufiventer*.

Our strategy was to assess whether haplotypes on opposite sides of a river(s) were reciprocally monophyletic with respect to one another and whether the pattern (if any) across species was temporally similar, indicating a shared evolutionary history with respect to divergence time.

For birds and lice, whole genomic DNA was extracted from tissue using the DNeasy tissue extraction kit (Qiagen Inc., Valencia, CA); louse specific protocols were used for lice (Cruickshank et al. 2001; Johnson and Clayton 2002). After DNA extraction, lice were mounted on slides and retained as vouchers. We used the polymerase chain reaction (PCR) to amplify portions of the cytochrome-*b* (cyt-*b*) gene for all avian samples; we also amplified the nuclear βact3 gene for *Bleda syndactyla* and the nuclear β-fibrinogen intron-5 gene (TGFβ2) for *Stiphrornis xanthogaster*. We amplified the cytochrome oxidase I (COI) gene for lice. PCR amplifications used published primers and protocols (Sorenson et al. 2004; Outlaw et al. 2007; Carling and Brumfield 2008; Light and Reed 2009). Automated sequencing was performed using BigDye (Applied Biosystems, Carlsbad, CA) and products were run on an ABI 377 sequencer at the University of Florida ICBR facility. We used SEQUENCHER, version 4.5 (Gene Codes, Ann Arbor, MI) to align sequences. To ensure the accuracy of amplification, we sequenced both heavy and light strands, and verified that sequence data were protein-coding. Sequences for louse taxa and those bird species for which genetic variation was evident (*Alethe*, *Bleda syndactyla*, *Illadopsis*, *Stiphrornis*) have been deposited on GenBank under accession numbers KC349953-KC349959 (lice) and KC355099-KC355178 (birds).

Uncorrected p-distances were examined for louse taxa and phylogenetic and other analyses were focused on avian taxa with genetic variation north and south of the Congo River (see Results: *Alethe castanea*, *Bleda syndactlya, Illadopsis rufipennis*, and *Stiphrornis xanthogaster*). We used MrModelTest (Nylander 2004) to determine the appropriate model of nucleotide substitution for each avian lineage (GTR+I, GTR+I+G, HKY+I, and HKY+G for *Alethe, Bleda syndactyla, Illadopsis,* and *Stiphrornis*, respectively). Sequence data for each understory species were analyzed using MRBAYES (Huelsenbeck and Ronquist 2001), where we initiated two runs of four Markov–chain Monte Carlo (MCMC) chains of 2 million generations each from a random starting tree, sampling every 100 generations. Each run resulted in 20,000 trees and converged on the same topology. The first 50,000 generations (5000 trees) from each analysis were removed as our “burn-in”, and the remaining 30,000 trees were used to create a majority rule consensus tree. Trees were rooted using mid-point rooting.

We used the program BEAST v1.6.1 (Drummond and Rambaut 2007) to estimate TMRCA within each species. We employed a lineage substitution rate of 0.0105 per site/million years using a relaxed, uncorrelated lognormal clock. This substitution rate translates to 2.1% per million years, and is generally applicable to the cyt-*b* gene in songbirds (Weir and Schluter 2008; Lerner et al. 2011). We also employed a normal distribution for this prior and assigned a standard deviation of 0.0013, which encompasses a slower (1.6%) and faster (2.53%) estimate calculated for songbird cyt-*b* substitution rates in other studies (Fleischer et al. 1998; Nabholz et al. 2009). A Yule process speciation prior was implemented in each analysis. Two separate MCMC analyses were run for 10,000,000 generations with parameters sampled every 1000 steps, and a 10% burn-in. Independent runs were combined using LogCombiner v.1.6.1 (Drummond and Rambaut 2007; Drummond et al. 2012). Tracer v.1.5 (Rambaut and Drummond 2007) was used to measure the effective sample size of each and calculate the mean and upper and lower bounds of the 95% highest posterior density interval (95% HPD) for divergence times. Tree topologies were assessed using TreeAnnotator v.1.7 (Drummond and Rambaut 2007; Drummond et al. 2012) and FigTree v.1.3.1 (Rambaut 2008).

*BEAST (Drummond and Rambaut 2007; Heled and Drummond 2010) was used to infer a “multi-species” tree (consisting of two taxa: “north” and “south” of the Congo River) from cyt-*b* gene trees obtained from *Alethe, Bleda syndactlya, Illadopsis,* and *Stiphrornis*. *BEAST was used to determine if the Congo River caused divergence within each of these bird species at approximately the same time. Specimens were identified as being collected from north or south of the Congo River and used to reconcile the evolutionary history of bird taxa in the Congo Basin. All analyses were run in BEAST v1.7 (Drummond and Rambaut 2007; Drummond et al. 2012) using the models of molecular evolution identified in the phylogenetic analyses (see above). Several analyses were run to reconcile the evolutionary history of these four species. Preliminary BEAST analyses resulted in the parameter ucld.stdev (the standard deviation of the uncorrelated lognormal relaxed clock) being close to 0 (suggesting that the avian cyt-*b* data are clocklike). We therefore performed analyses enforcing a strict clock as well as a relaxed, uncorrelated lognormal clock for the substitution rate for comparison. We also varied the speciation prior, using a Yule process as well as a speciation birth-death process speciation prior. Relative evolutionary rates were estimated among the three gene trees (*Bleda syndactyla, Illadopsis,* and *Stiphrornis*) by setting the prior of the rate for *Alethe* at 1.0. For each analysis, two separate MCMC runs were run for at least 60 million generations with parameters sampled every 1000 steps, and incorporating a 10% burn-in. Combining of MCMC runs, and assessment of parameters and trees were performed as above.

MsBayes (Hickerson et al. 2006, 2007) was used to test for simultaneous divergence times in *Alethe*, *Bleda syndactyla*, *Illadopsis*, and *Stiphrornis*. Individuals from each species were identified as being collected from north or south of the Congo River and data from all four species were considered in combination and number of divergences was evaluated. One million simulations were drawn from the hyper-prior and the hyper-posterior was constructed from 1000 samples (tolerance = 0.005) using the hierarchical approximate Bayesian computation acceptance/rejection algorithm.

## Results

We generated mitochondrial sequence data for 10 species of birds based on our field sampling across the Congo River and its tributaries (Fig. 1, Table 1). For six of these species, some of which are (*Neocossyphus poensis, Bleda ugandae*) and some of which are not (*Terpsiphone rufiventer*, *Criniger calurus, Myioceyx leconteii, Smithornis rufolateralis*) understory specialists, we found no evidence of across river genetic diversification; we did not analyze these species further. We found no variation in either the nuclear βact3 gene across *Bleda syndactyla* samples, or nuclear TGFβ2 gene for *Stiphrornis xanthogaster*.

**Figure 2:**
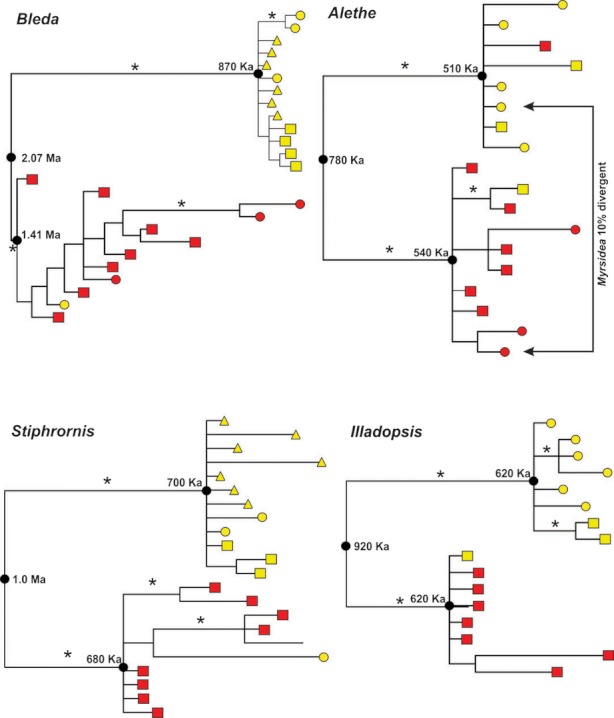
Phylograms of each species, where sampling site replaces individual. Asterisks indicate posterior probabilities ≥0.95. Phylograms are mid-point rooted. Time to most recent common ancestor is shown at the base of each clade; associated 95% HPD values are presented in Table 2. Percent sequence divergence is shown for *Myrsidea* lice parasitizing individuals of *Alethe*.

Within each understory avian lineage for which we found genetic variation across the Congo (*Alethe castanea*, *Bleda syndactlya, Illadopsis rufipennis*, and *Stiphrornis xanthogaster*), Bayesian analysis resulted in two highly supported clades. These clades consist almost entirely of haplotypes from birds collected from north of the Congo River versus haplotypes of birds collected from south of the river (Fig. 2). In each species, there is just one instance of a bird collected on the north bank having a southern bank haplotype; the reverse is true in *Alethe*. We found no evidence to suggest divergence across Congo River tributaries (Fig. 2). Further, for *Bleda syndactyla* and *Stiphrornis xanthogaster*, we found no sorting of haplotypes between north bank samples and those collected to the east (triangles in Figs. 1 and 2). This suggests that the genetic variation evident across the Congo River (ca. 50 km near Kisangani) is not a function of distance effects.

Estimates of time to most recent common ancestor (TMRCA) in each avian lineage (Fig. 2, Table 2) were consistent with latest Pliocene (*Bleda syndactyla*) or Pleistocene origins (other lineages). With the exception of southern bank *Bleda syndactyla* haplotypes, northern and southern clades across lineages have generally similar TMRCA (Fig. 2, Table 2). These TMRCA are indicative of a shared evolutionary history.

**Table 2:** Divergence time estimates (BEAST) and relative root height estimates north and south of the Congo River (*BEAST) for the four understory bird species showing genetic differentiation. Basal divergence (time to most recent comment ancestor; TMRCA) for each species represents the split between clades from the northern and southern sides of the Congo River; TMRCA for north and south clades also are shown. *BEAST results are for those using a strict clock, Yule process speciation prior, and setting the prior evolutionary rate for *Alethe* at 1.0

	TMRCA Mean (95% HPD) in millions of years	*BEAST Root Height Estimates
*Alethe castanea*		
Basal Divergence	0.78 (0.38, 1.25)	0.013 (0.007, 0.019)
North	0.51 (0.18, 0.86)	
South	0.54 (0.22, 0.90)	
*Bleda syndactyla*		
Basal Divergence	2.07 (1.13, 3.15)	0.019 (0.007, 0.033)
North	0.87 (0.32, 1.51)	
South	1.41 (0.77, 2.13)	
*Illadopsis rufipennis*		
Basal Divergence	0.92 (0.46, 1.42)	0.023 (0.009, 0.041)
North	0.62 (0.26, 1.03)	
South	0.62 (0.25, 1.02)	
*Stiphrornis xanthogaster*		
Basal Divergence	1.00 (0.55, 1.51)	0.014 (0.006, 0.024)
North	0.70 (0.34, 1.10)	
South	0.68 (0.31, 1.09)	

**Figure 3:**
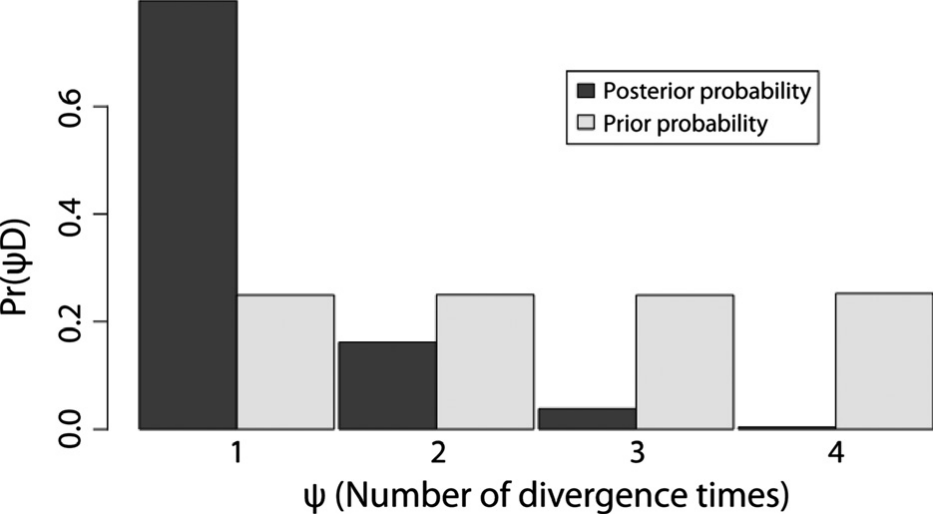
MsBayes histogram of the prior and posterior distribution for number of divergence times (ψ) for four bird species (*Alethe, Bleda syndactyla, Illadopsis,* and *Stiphrornis*) across the Congo River.

MsBayes failed to reject the null hypothesis of simultaneous divergence of the four understory species pairs across the Congo River (Fig. 3), despite the estimated divergence time for *Bleda syndactyla* being about twice the other species (Fig. 2). In all analyses, the posterior estimate of the number of possible divergence times (ψ) was not significantly different from one and values of Ω were low, both of which support a single divergence event. Similarly, regardless of the type of *BEAST analysis, root height estimates for *Alethe*, *Bleda syndactlya, Illadopsis*, and *Stiphrornis* were nearly identical with overlapping 95% Higher Posterior Density intervals (HPD: Table 2), indicating approximately simultaneous divergence times north and south of the Congo River. Estimates of relative evolutionary rates revealed similar mutation rates among *Alethe*, *Illadopsis*, and *Striphornis*, whereas mutation rates in *Bleda syndactyla* were approximately 2.5 times faster (mean = 2.68; 95% HPD: 1.07–4.68), consistent with the older estimated age for this species (Fig. 2, Table 2).

With respect to lice, *Myrsidea* species (Phthiraptera: Amblycera) parasitizing *Alethe castanea* individuals from north and south of the Congo River were 10% divergent in their DNA, whereas their hosts were just 2.2% divergent (uncorrected *p*-distances). We also found across-Congo divergences of 4% in a *Myrsidea* species taken from another bird species (*Terpsiphone rufiventer*). These across-river divergences contrasts with a lack of genetic divergence in another louse species (*Sturnidoecus* sp.; Phthiraptera: Ischnocera), also parasitizing *T. rufiventer*. Notably, *T. rufiventer* occupies intermediate levels of a variety of forest types, and shows no genetic divergence across the Congo.

## Discussion

Our results provide clear evidence of mitochondrial genetic variation across the Congo River. We found no variation in the βact3 nuclear gene for *Bleda syndactyla*. Nor did we find variation in the nuclear TGFβ2 gene for *Stiphrornis xanthogaster*; variation in this gene appears useful only at the interspecific level, where it supports a sister-relationship between *xanthogaster* and *sanghensis*, relative to other *Stiphornis* species (G. Voelker, unpubl. data). The divergence in mitochondrial data is in the realm of 2*N*_*e*_, suggesting that no nuclear gene variation should be expected (Palumbi et al. 2001; Zink and Barrowclough 2008). Therefore, we did not screen additional nuclear genes, and instead focus on mitochondrial DNA as a leading indicator of differentiated taxa (Zink and Barrowclough 2008; Barrowclough and Zink 2009); importantly, reasonable estimates of divergence dates exist for these data (Weir and Schluter 2008; Lerner et al. 2011).

The Congo River appears to serve as a barrier to gene flow in four species of Afrotropical understory birds. Our results thus show limited support for Wallace's (Wallace 1852) RBH. Furthermore, genetic divergences across the four avian species are indicative of a shared evolutionary history that coincides temporally with the formation of the Congo River (Figs. 2 and 3). Haplotypes are not strictly north bank versus south bank in each species, and this could be the result either of limited across-river migration or of incomplete lineage sorting across the river. The TMRCA for each clade in all four species is similar and this temporal uniformity suggests that north bank and south bank clades have been genetically isolated for a long period. Thus, it is doubtful that incomplete lineage sorting would be temporally consistent across four species. Instead, we hypothesize that limited migration can be inferred to explain the presence of north or south bank haplotypes on the opposite side of the river. It is important to note that the Congo River is clearly not serving as a generalized avian barrier; we found genetic variation in only four of the 10 species examined.

For habitat generalists, the lack of variation was not particularly surprising to us. However, we were surprised to find that not all understory specialists harbored genetic variation; two of the six understory specialists examined in this study (*Neocossyphus* and *Bleda ugandae*) showed no genetic variation across the Congo River. In addition to shared habitat, all understory specialists we surveyed are species that follow and prey on army ant swarms (del Hoyo et al. 2005, 2007). The shared habitat and foraging ecology of these understory specialists had led us to expect similar genetic variation. For *Neocossyphus*, the lack of variation could be due to just a single sample having been collected from the north bank of the Congo River (Table 1). This sample may then simply reflect incomplete lineage sorting or some level of across river movement as we see in other species (Fig. 2). However, for *Bleda ugandae*, the sampling is more balanced (Table 1) and we cannot explain why this species does not harbor some level of genetic variation as compared with its ecologically similar counterparts. Overall, due to the relatively small and geographically restricted sample sizes of this study, we are unable to make any generalizations about the role the Congo River as a barrier to gene flow for other taxa in the Congo Basin.

However, the Congo River appears to serve as a barrier to gene flow in two of the three avian parasites for which we had samples that allowed cross-river comparisons. This discrepancy in the effect of the Congo River as a genetic barrier is in part related to the biology of the different louse groups. Amblyceran lice (e.g., *Myrsidea*) feed on host body fluids (blood), whereas ischnoceran lice (e.g., *Sturnidoecus*) feed on feathers. The feeding behavior of amblycerans increases exposure to the host's immune system (Marshall 1981), which may promote selection toward host specific forms and accelerate their genetic variation relative to Ischnocera. Furthermore, amblyceran lice are found among the body feathers of their hosts, whereas ischnoceran lice are found in the wing feathers. Body lice are competitively superior to wing lice, and in response wing lice will actively practice host-switches via phoresis, the mechanical transmission among hosts via hippoboscid flies (Marshall 1981; Harbison et al. 2008, 2009; Harbison and Clayton 2011). The ability of ischnoceran wing lice to transfer among hosts can result in a reduction in host specificity and lower levels of intraspecific variability (Keirans 1975; Bueter et al. 2009; Stefka et al. 2011). Thus, for lice parasitizing birds from opposite banks of the Congo River, a combination of feeding behavior and inability to disperse may be leading to higher host specificity and genetic variation in body lice. Conversely, phoresis in wing lice may be facilitating host-switches, resulting in little to no genetic differentiation among lice on birds from opposite banks of the Congo River. Host ecology and dispersal also affects these genetic differences in lice: understory birds, such as *Alethe castanea* that are genetically differentiated on opposite banks of the Congo River harbor genetically differentiated lice. In contrast, birds, such as *Terpsiphone rufiventer* that are lacking genetic variation on opposite banks of the Congo River are parasitized by lice having little to no genetic variation.

From our results, it is evident that potentially extensive cryptic diversity is being harbored in Afrotropical forests. With respect to birds, these results run counter to the assertion of Fjeldså and Lovett (Fjeldså and Lovett 1997) that Afrotropical forests are museums where little in the way of evolutionary diversification occurs. Our results also suggest that a lack of plumage variation should not be considered indicative of a lack of genetic variation. Indeed, three of our understory taxa were explicitly categorized (along with 105 other avian taxa) as “uninformative” due to lack of plumage variation (Mayr and O'Hara 1986), as was another species (*Hylia prasina*) recently found to harbor extensive geographically structured genetic diversity across Afrotropical forests (Marks 2010). That each of these lineages shows genetic variation and largely contemporaneous divergence times suggests that broad additional screening of “uninformative” taxa may shed new light on both the RBH and the Pleistocene Forest Refuge Hypothesis as possible drivers of Afrotropical divergences (Wallace 1852; Mayr and O'Hara 1986). And while these hypotheses are not necessarily mutually exclusive (e.g., taxa historically segregated in refugia may now meet at river barriers), divergence dating may allow us to more accurately determine whether broad patterns are the result of single or multiple events. This seems a particularly relevant point with respect to the Forest Refuge Hypothesis, which through time has been misrepresented as being solely linked to the Pleistocene (Haffer 1997).

Our analyses provide the first intraspecific molecular assessments of the Riverine Barrier Hypothesis for birds and their lice distributed in the Afrotropics. Because our results are based on data from a very small area of the Congo Basin, extensive cryptic diversity is likely being harbored in Afrotropical forests. More broadly, the possibility of high levels of cryptic diversity in the Afrotropics is important when considering that macroecological studies use distributional information in range maps (Hansen et al. 2007) as their fundamental data points (Jetz et al. 2004), and that results of these studies inform conservation prioritization. Due in part to the perpetuation of Afrotropical forests as museums (Brooks et al. 2006) labeled the Congo Basin as an area with low vulnerability. This implies that habitat loss in the Congo Basin would not result in a significant loss of biodiversity because most species found there are widespread, and suitable similar habitat exists in other parts of the Afrotropical forest region (Marks 2010). That we find strong genetic diversification in birds and their lice within a small part of the Congo Basin forest indicates that we have perhaps grossly underestimated genetic diversity, and thus levels of vulnerability, in the Afrotropics.
